# Human Placental Trophoblasts Are Resistant to *Trypanosoma cruzi* Infection in a 3D-Culture Model of the Maternal-Fetal Interface

**DOI:** 10.3389/fmicb.2021.626370

**Published:** 2021-03-04

**Authors:** Erica Silberstein, Kwang Sik Kim, David Acosta, Alain Debrabant

**Affiliations:** ^1^Laboratory of Emerging Pathogens, Office of Blood Research and Review, Center for Biologics Evaluation and Research, Food and Drug Administration, Silver Spring, MD, United States; ^2^Division of Pediatric Infectious Diseases, Johns Hopkins University School of Medicine, Baltimore, MD, United States

**Keywords:** chagas disease, congenital infection, 3D culture system, human trophoblasts, *Trypanosoma cruzi*

## Abstract

*Trypanosoma cruzi* (*T. cruzi*), the etiological agent of Chagas Disease (CD), is transmitted to humans by infected kissing bugs, blood transfusion, organ transplantation, and from mother-to-child. Congenital transmission is now considered an important route of CD spread in non-endemic countries where no routine testing of pregnant women for the disease is implemented. The main cellular mechanisms that lead to fetal infection by *T. cruzi*, despite the presence of a placental barrier, remain unclear. Mother-to-child transmission most likely occurs when bloodstream trypomastigotes reach the placental intervillous space and interact with the large cellular surface provided by the syncytioptrophoblasts. These highly specialized cells not only function as a physical obstacle between mother and fetus, but also modulate immune responses against pathogen infections. To overcome the limitations associated with the use of human fetal tissues, we employed a three-dimensional (3D) cell culture model to recreate the human placenta environment. In this system, the trophoblast-derived JEG-3 cell line is co-cultured with human brain microvascular endothelial cells attached to microcarrier beads in a rotating bioreactor. Here, we report that 3D culture of JEG-3/HBMEC spheroids promote JEG-3 cells differentiation revealed by the formation of syncytia and production of β human chorionic gonadotropin and human placental lactogen (hPL). Under these growth conditions, we demonstrate that 3D-grown JEG-3 cells have reduced susceptibility to *T. cruzi* infection compared to JEG-3 cells grown in conventional tissue culture flasks. We also show that 3D-cultured JEG-3 cells release paracrine factors in the supernatant that prevent *T. cruzi* infection of non-trophoblastic cell lines. Our *in vitro* model of *T. cruzi* vertical transmission may help better understand the molecular processes by which parasites bypass the human placental barrier and could be exploited to evaluate therapeutics to reduce congenital CD.

## Introduction

Chagas disease (CD) caused by *Trypanosoma cruzi (T. cruzi)*, is a major public health neglected tropical disease that has spread globally through migration of infected individuals from endemic countries of Latin America to the United States, Europe, Japan, and Australia (Requena-Mendez et al., 2015; Perez-Molina and Molina, 2018; Lidani et al., 2019).

In endemic countries, *T. cruzi* is transmitted to humans by infected triatomine bugs. Other modes of parasite transmission include blood transfusion, organ transplantation, and from mother-to-child. *T. cruzi* congenital infection is considered a major route of CD spread in non-endemic areas (Carlier et al., 2019; Rios et al., 2020). Recent studies indicate that 40,000 *T. cruzi*-infected women of childbearing age live the United States where the estimated vertical transmission rate is 1–5% (Messenger and Bern, 2018; Edwards et al., 2019). Although most infected newborns are asymptomatic at birth, they are frequently born premature and with low birthweight. In some cases, congenitally infected babies may suffer from severe symptoms that can lead to death (Torres-Vargas et al., 2018). There is no vaccine to prevent CD and the available anti-parasitic drugs (benznidazole and nifurtimox) are contraindicated for pregnant women. Yet, the efficacy of benznidazole treatment in infants younger than 1 year ranges from 90 to 100% and consequently early diagnosis and therapeutic interventions can prevent the development of chronic CD later in life (Perez-Zetune et al., 2020).

As the main link between the mother and the fetus, the placenta facilitates the exchange of metabolites, produces hormones, and modulates immune responses to prevent vertical transmission of pathogens (Arora et al., 2017; Ander et al., 2019; Kemmerling et al., 2019). The human placenta is composed by free-floating chorionic villous trees covered by a single layer of polarized multinucleated syncytiotrophoblasts (STs) of fetal origin. Maternal blood surrounds the STs, which display a dense brush border on their apical surfaces forming a physical barrier that protects the fetus from parasites, viruses and other pathogens.

Because trans-placental infection occurs, *T. cruzi* trypomastigotes present in maternal blood must be able to counter the ST barrier to reach the fetus. The mechanisms by which parasites travel across the placenta remain to be elucidated. The development of laboratory animal models that can reproduce the complexities of congenital infections continues to be a challenge (Barrila et al., 2018) as the placental architecture of commonly used species such as mice and rats is morphologically different from the human placenta (Cencig et al., 2013; Barrila et al., 2018; Torres-Vargas et al., 2018; Ander et al., 2019). Thus, results from small animal studies may not be directly applicable to human congenital transmission.

While many investigators have worked with primary human trophoblasts (Medina et al., 2018; Triquell et al., 2018) and placental chorionic villi explants (Duaso et al., 2010; Castillo et al., 2017a; Liempi et al., 2020) to explore host-pathogen interactions, such models are not genetically tractable and procurement of human fetal-derived tissues is highly restricted (Yoshizawa, 2013). Flat two-dimensional (2D) cell cultures of trophoblasts have been widely used to study infectious disease intracellular mechanisms, even though they lack many essential characteristics of the native host microenvironment (Droguett et al., 2017; Medina et al., 2018). For instance, cell lines derived from choriocarcinomas express trophoblast specific-markers but do not form syncytia and therefore, fail to mirror the biology of the continuous STs layer (Orendi et al., 2010). In contrast, three-dimensional (3D) culture systems offer opportunities for investigations in a more physiologically relevant milieu because they can mimic tissue architecture, multicellular complexity, oxygen exchange, nutrient transport, and biomechanical forces (e. g., fluid shear) (Barrila et al., 2018).

In this report, we used a 3D cell culture of human trophoblasts to reproduce the maternal-fetal interface (McConkey et al., 2016) and study *T. cruzi* infection of the human placenta in an environment resembling a natural infection. When cultured in 3D, JEG-3 cells (3D JEG-3) formed syncytia, and produced placental-specific hormones. Here, we provide evidence that fully differentiated 3D JEG-3 cells become resistant to *T. cruzi* infection, illustrating the advantage of this *in vitro* placenta model for studying key features of the parasite interactions with its host and highlights its potential use in the evaluation of therapeutics to reduce congenital CD.

## Materials and Methods

### Mammalian Cell Culture

JEG-3 cells [ATCC^®^ HTB-36^TM^, (Kohler and Bridson, 1971); American Type Culture Collection, VA] were cultured in conventional tissue culture flasks (2D culture conditions) in Eagle’s Minimum Essential Medium (EMEM; Life Technologies, CA) containing L-glutamine, 1% penicillin/streptomycin and 10% fetal bovine serum (FBS). Human brain microvascular endothelial cells (HBMECs) were cultured in Roswell Park Memorial Institute *1640 Medium* (*RPMI 1640*, Life Technologies, CA) containing L-glutamine, 1% penicillin/streptomycin, 10% FBS, 10% Nu-Serum^TM^ IV Growth Medium Supplement (Corning, NY), sodium pyruvate, non−essential amino acids and MEM vitamins as described (Coyne et al., 2007). LLC-MK2 original cells (ATCC^®^ CCL-7^TM^; American Type Culture Collection, VA) were cultured in Dulbecco’s Modified Eagle’s Medium (DMEM: Life Technologies, CA) containing L-glutamine, 1% penicillin/streptomycin and 10% FBS (Hull et al., 1962). All cell lines were grown in a humid atmosphere containing 5% CO_2_ at 37°C. Cell counts were determined using a Cellometer K2 Fluorescent Viability Cell Counter (Nexcelom Bioscience, MA).

### Three-Dimensional (3D) Rotating Wall Vessel (RWV) Bioreactor Cultures

**FIGURE 1:**
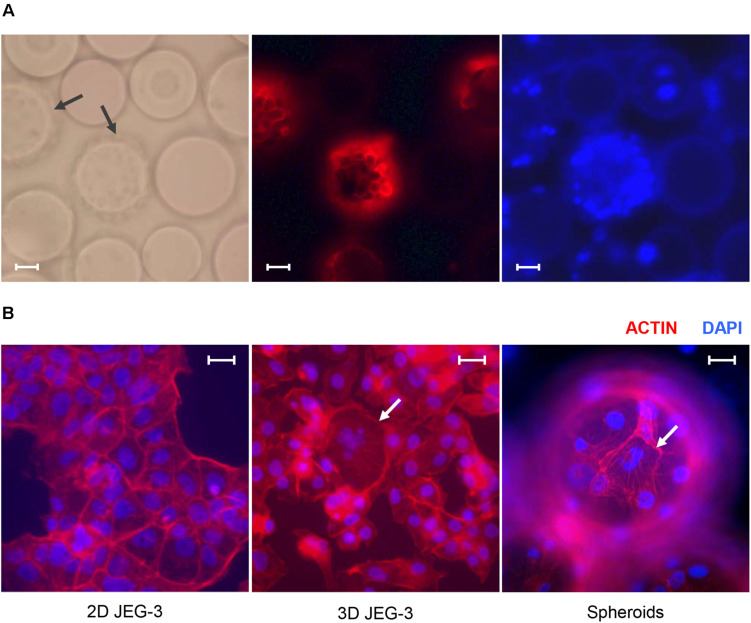
JEG-3 cells grown in the RWV bioreactor fuse to form syncytia. **(A)** Micrographs of JEG-3/HBMEC spheroids cultured for 20 days. Cell membranes were stained with Texas Red^®^ 1,2-dihexadecanoyl-*sn*-glycero-3-phosphoethanolamine, triethylammonium salt (Texas Red^®^ DHPE, red). *Left:* bright-field. *Center*: Texas Red^®^ DHPE. *Right:* 4’, 6-diamindino-2-phenylindole (DAPI). Arrows point to cell coated beads (spheroids). **(B)** Staining of actin filaments in monolayers of 2D JEG-3 cells (left) or 3D-cultured cells, either detached from beads (center) or on 3D-grown spheroids (right). Cells were fixed/permeabilized and stained with Alexa Fluor^®^ 680 phalloidin (red). Arrows point to syncytia. Slides were mounted with Vectashield mounting medium containing DAPI (blue) to visualize nuclei. Fluorescent micrographs are representative of three independent experiments and were acquired on a Keyene BZ-9000 fluorescence microscope. Scale bars: 50 μm in **(A)** and 30 μm in **(B)**.

**FIGURE 2:**
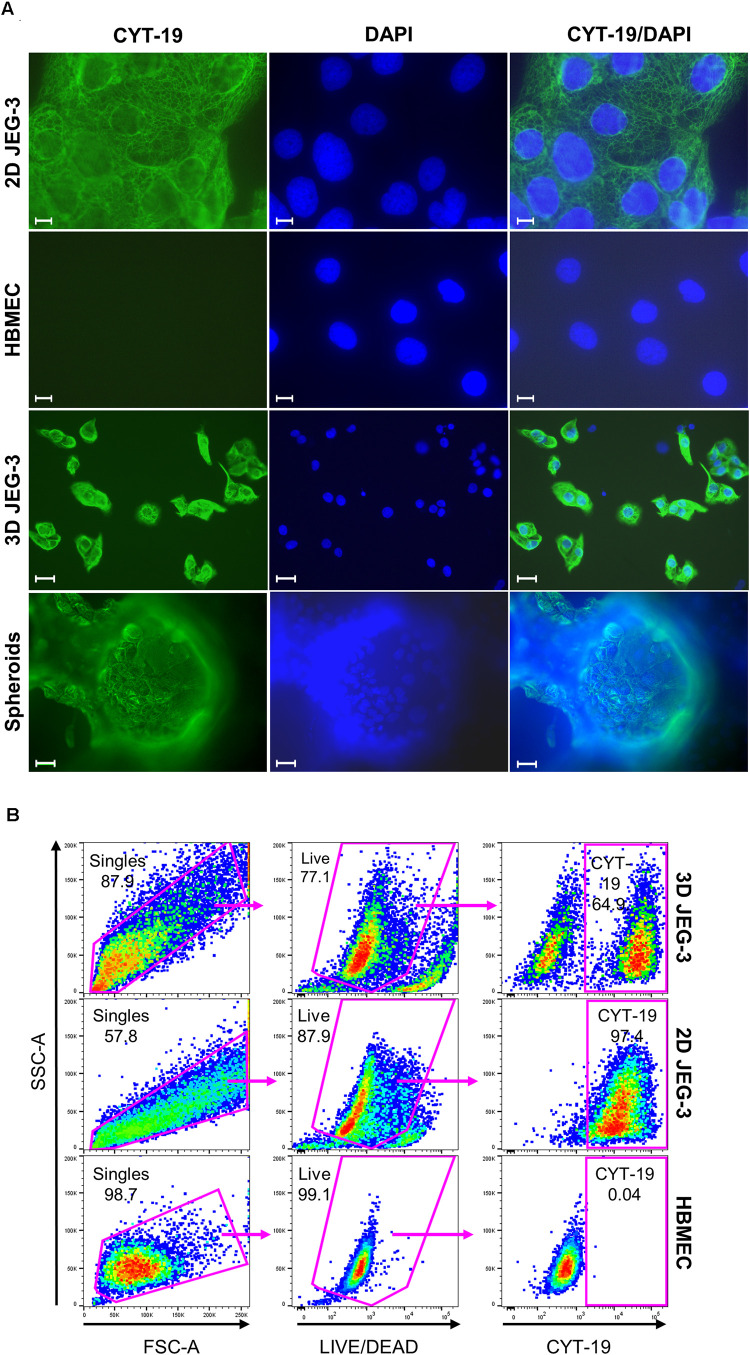
JEG-3 cells but not HBMECs express cytokeratin-19. **(A)** Immunostaining of 2D JEG-3 cells, HBMECs, 3D-cultured cells detached from beads, and spheroids, as indicated. Cell monolayers and spheroids were fixed/permeabilized and stained with rabbit anti-CYT-19 followed by Alexa Fluor 488 anti-rabbit IgG (H + L) antibodies (green). Slides were mounted with Vectashield mounting medium containing DAPI to visualize nuclei (blue). Fluorescent micrographs were acquired on a Keyene BZ-9000 fluorescence microscope. Scale bars: 10 μm (2D JEG-3 and HBMEC panels); 30 μm (3D JEG-3 cells detached from beads and spheroids). **(B)** Flow cytometry analysis for CYT-19 expression in 3D-cultured cells after detachment from beads, 2D JEG-3 and HBMEC cells, as indicated. Gating scheme to identify singles and exclude dead cells based on LIVE/DEAD^TM^ violet dye staining is shown. Data are representative of two independent experiments.

HBMECs grown in conventional flasks were harvested using 0.05% trypsin/EDTA. Cells were washed, and resuspended in GTSF-2 media (Lelkes et al., 1997) containing 1% penicillin/streptomycin and supplemented with insulin-transferrin-sodium selenite (Gibco, CA), 10% FBS, HEPES, sodium bicarbonate (21.2 mM), peptone (0.6%), galactose (1.4 mM), fructose (0.7 mM), and glucose (5.6 mM). 7 × 10^5^ HBMECs resuspended in GTSF-2 media were mixed with 50 mg collagen-coated beads (Cytodex-3 beads; Sigma, MO). After a 30 min incubation at 37°C, the cell/bead slurry was transferred to a disposable slow-turning lateral 10 ml culture vessel (Synthecon, TX) and loaded onto the rotor. Cells were grown for 3 days at 14 rpm. Next, 10^6^ JEG-3 cells were added to the vessel and incubated without rotation for 30 min at 37°C. Finally, the culture continued under rotation (14 rpm) for 17 days. The medium was replaced every 24–48 h throughout the culture period (McConkey et al., 2016). To harvest 3D-cultured cells, cell-coated beads (spheroids) were transferred from the RWV bioreactor’s vessel to 50 ml tubes. Next, spheroids were either transferred directly to 96-well plates (infection experiments described in Figure 4A and Supplementary Figure 5A), or cells were detached from the beads for immunofluorescence or flow cytometry analysis (Figures 1B, 2, 4B–D). To detach 3D-cultured cells, spheroids were washed with PBS and incubated with TryPLE Express (Gibco, CA) for 30 min at room temperature (RT). After incubation, the cell/bead slurry was filtered through a 100 μm cell strainer and cells were collected by centrifugation at 1,200 rpm for 10 min at 4°C.

### Cell Viability Assay

Live-cell numbers were determined using the CellTiter-Fluor^TM^ cell viability assay as per manufacturer’s instructions (Promega, WI). Briefly, LLC-MK2, HBMECs and 2D JEG-3 cells (seeded at a density of 1 × 10^4^ cells/well) were grown directly in white wall/clear bottom 96-well plates. For 3D-grown cultures, 100 μl aliquots of spheroids suspension (collected from the RWV bioreactor’s vessel) were transferred to white wall/clear bottom 96-well plates. Next, 100 μl of CellTiter-Fluor^TM^ Reagent was added to the wells and incubated for 30 min at 37°C. Fluorescence [proportional to the number of live cells (Niles et al., 2007)] was measured in relative fluorescence units (RFU) at an excitation wavelength of 380 nm and an emission wavelength of 505 nm, using a SpectraMax M5 microplate reader (Molecular Devices, CA). The number of live cells was determined by extrapolation from a standard curve prepared with JEG-3 or HBMEC cells.

### *Trypanosoma cruzi* Propagation and Infection

Trypomastigotes of the *T. cruzi* strain Colombiana expressing nanoluciferase [TcCOL-NLuc, (Silberstein et al., 2018)] were propagated in LLC-MK2 cells (Hull et al., 1962). The number of live parasites in culture supernatants was determined using a Cellometer K2 Fluorescent Viability Cell Counter (Nexcelom Bioscience, MA).

Infections of HBMEC, 2D JEG-3 and LLC-MK2 cells were done in 96-well plates, with cells plated at a density of 1 × 10^4^ cells/well. For cultures grown in 3D, spheroids were removed from the RWV bioreactor’s vessel and transferred to 96-well plates (100 μl/well). After determining the number of viable cells with the CellTiter-Fluor^TM^ cell viability assay (as explained above), cells were infected with TcCOL-NLuc trypomastigotes at a ratio of 5 parasites/live cell. Sixteen hours post-infection, conventional and 3D cultures were washed to remove non-internalized parasites and subsequently incubated for 3 days. All infections were performed under static conditions in 96-well plates with exception of the flow cytometry experiment shown in Figure 4B, in which 3D-grown spheroids, were infected at a ratio of 5 parasites/live cell (based on the cell number determined from a 100 μl aliquot collected from the vessel), and then cultured in the RWV bioreactor under rotation.

### Immunofluorescence Microscopy

For membrane staining of live cells cultured in 3D, 200 μl of spheroids collected from the RWV bioreactor’s vessel were transferred to 1.5 ml tubes, allowed to precipitate and washed twice with PBS. Cells were next stained with 1 mM Texas Red 1,2-dihexadecanoyl-sn-glycero-3-phosphoethanolamine, triethylammonium salt (Texas Red DHPE; Invitrogen, CA) for 15 min at RT, transferred to Permanox^TM^ 8-well slides (Thermo Fisher Scientific, MA) and fixed with 4% paraformaldehyde (PFA).

For intracellular staining of actin and cytokeratin-19 (CYT-19), 2D JEG-3 and HBMEC cells (grown in 8-well chamber slides), spheroids (100 μl collected from RWV bioreactor and transferred to 8-well chamber slides), or 3D JEG-3 cells (detached from the beads using TryPLE Express and seeded into 8-well chamber slides), were fixed with 4% PFA and permeabilized in Perm/Wash^TM^ Buffer (BD Biosciences, NJ) for 15 min at RT. Samples were next stained with the following fluorescent dyes or antibodies diluted in PBS/1% bovine serum albumin (BSA): 20 μM solution of Alexa Fluor 680 phalloidin (Invitrogen, CA) and rabbit anti-CYT-19 antibodies (1/200 dilution; Abcam, MA) for 1 h at RT. After incubation, cells were washed with PBS and stained with Alexa Fluor 488 anti-rabbit IgG (H + L) antibodies (1/1,000 dilution; Invitrogen, CA). Slides were mounted using Vectashield mounting medium (Vector Laboratories, Burlingame, CA) containing 4’, 6-diamindino-2-phenylindole (DAPI) and observed by microscopy. Phase contrast and fluorescence micrographs were acquired on a Keyene BZ-9000 fluorescence microscope at 40 or 100X magnification.

### Human β Chorionic Gonadotrophin Assay

Secretion of human β chorionic gonadotrophin (β hCG) in cell culture supernatants was measured using the HCG ELISA kit following the manufacturer’s protocol (Abnova, CA). Culture medium of 3D HBMEC, 3D JEG-3 (grown in the RWV bioreactor) and 2D JEG-3 cells (grown in T75 flasks) was collected at 3, 8, 15, 17, 20, and 21 days of culture (depending on the cell type) and spun down at 3,500 rpm for 5 min to remove any cellular debris. In all cases, the medium was changed 24 h before supernatant collection for ELISA. Levels of β hCG were extrapolated from a standard curve and expressed as milli-international units per milliliter (mIU/ml).

### RNA Isolation an qRT-PCR

Total RNA was extracted from harvested 3D-JEG-3, 2D JEG-3, and 3D HBMEC cells with the PureLink^TM^ RNA mini kit (Invitrogen, CA) and on-column DNase treatment. RNA integrity and quality were analyzed in a 2100 Bioanalyzer system (Agilent Technologies Inc., CA). Following purification, total RNA was reverse-transcribed into cDNA using the iScript cDNA synthesis kit (Bio-Rad Laboratories, CA) and 0.5 μg of RNA template. qPCR was carried out using IQ SYBR Green Supermix (Bio-Rad Laboratories, CA) in a CFX96 touch real-time PCR detection system (Bio-Rad Laboratories, CA) and the data were analyzed with the CFX manager software. Gene expression was determined by the 2^–ΔΔ*Ct*^ method with mRNA levels normalized to β actin (Livak and Schmittgen, 2001). Primer sequences were as follows: human placental lactogen (hPL) [5’-CATGACTCCCAGACCTCC TTC-3’; 5’-TGCGGAGCAGCTCTAGATTG-3’ (Kawashima et al., 2014)]; endothelial nitric oxide synthase (eNOS) [5’-GTGGCTGTCTGCATGGACCT-3’; 5’-CCACGATGGTGACTTTGGCT-3’ (Salvolini et al., 2019)], and β actin [5’-TGACATTAAGGAGAAGCTGTGCTAC-3’; 5’- ACTTCATGATGGAGTTGAAGGTAGT-3’ (Rydbirk et al., 2016)].

### Labeling of *T. cruzi* Parasites With Fluorescent Dye

Culture-derived TcCOL-NLuc trypomastigotes were labeled with CellTrace^TM^ Far Red following manufacturer’s instructions (Invitrogen, CA). Briefly, 10^6^ parasites/ml were incubated for 20 min at RT with 1 μM CellTrace^TM^ Far Red dye protected from light. The excess of dye was quenched by the addition of 5 volumes of DMEM/2% FBS. Next, cells were centrifuged at 3,500 rpm for 10 min, resuspended in fresh medium and used for infection of cell cultures.

### Flow Cytometry Analysis

2D and 3D cultured JEG-3 cells were infected with CellTrace^TM^ Far Red-labeled TcCOL-NLuc trypomastigotes at a ratio of 5 parasites per live cell. After 16 h of incubation at 37°C, cultures were washed 3 times with PBS to remove non-internalized parasites. Cells were next detached from flasks or microcarrier beads by incubation with TryPLE Express and stained with the LIVE/DEAD^TM^ fixable violet stain kit according to manufacturer’s protocols (Invitrogen, CA). Following a 30 min incubation at RT, cells were washed with PBS/1% BSA and fixed using the Cytofix/Cytoperm Kit (BD Biosciences, NJ). Intracellular staining was conducted with Alexa Fluor 488 anti-human CYT-19 (Invitrogen, CA) diluted 1/40 in Perm/Wash^TM^ Buffer (BD Biosciences, NJ) for 30 min at 4°C. Cells were next washed twice with Perm/Wash^TM^ Buffer, resuspended in Stain Buffer (BD Biosciences, NJ) and acquired on an LSR Fortessa X20 (BD Biosciences, NJ). Data were analyzed with FlowJo software version 10. ArC amine-reactive and UltraComp eBeads Plus compensation beads (Invitrogen, CA) were used as positive and negative staining controls. Dead cells were excluded from the analysis based on staining with LIVE/DEAD^TM^ Fixable Violet dead cell stain. JEG-3 cells were identified as uniquely expressing CYT-19. *T. cruzi* infected cells were identified using the CellTrace^TM^ Far Red label. Analysis of non-infected 2D and 3D JEG-3 cells detached from the beads was done following similar procedures for the staining of dead cells and intracellular CYT-19.

### Nanoluciferase Assay for *Trypanosoma cruzi* Replication

HBMECs, LLC-MK2, and 2D JEG-3 cells were plated in 96-well strip white plates (clear bottom) and grown to 50% confluency. 3D JEG-3 spheroids were collected after 20 days in culture, and 100 μl of the suspension was transferred to each well. Live-cell numbers were determined using the CellTiter-Fluor^TM^ Reagent as explained above. Cells were infected with TcCOL-NLuc trypomastigotes at a ratio of 5 parasites per live cell. After 16 h (*T* = 0) of incubation at 37°C, cultures were washed three times with PBS to remove non-internalized parasites, and the infection proceeded for three additional days.

Parasite growth was determined at 0 (*T* = 0), 1 (*T* = 1), and 3 (*T* = 3) days post-infection by measuring nanoluciferase (NLuc) activity per live cell. First, 100 μl of CellTiter-Fluor^TM^ Reagent was added to wells containing cells or spheroids, and incubated for 30 min at 37°C. After determining the relative fluorescence units (RFU), the reagent was discarded, and samples were washed twice with PBS. Finally, NLuc activity was assessed by addition of 25 μl of Nano-Glo Live Cell Reagent (Promega, WI), containing dilution buffer and furimazine. End-point luminescence readings (relative light units, RLU) were taken in a Spectra Max, M5 microplate reader (Molecular Devices, CA). Nanoluciferase activity (RLU) was normalized within each well to live-cell numbers (RFU) and plotted over time.

### Conditioned Medium Preparation and *T. cruzi* Infectivity Assay in Non-trophoblastic Cell Lines

Conditioned medium (CM) from 3D JEG-3 cells (3D JEG-3 CM) was collected every 48 h, from days 15 to 21 of culture in the RWV bioreactor. For preparation of 2D JEG-3 cells conditioned medium (2D JEG-3 CM), cells were grown to confluency in T75 flasks and culture supernatants were collected over the course of 48 h. After centrifugation (3,500 rpm for 5 min) to remove cell debris, the conditioned media were diluted to 50% using their respective fresh culture media.

For infectivity assays, LLC-MK2 and HBMEC cells seeded in 96-well strip white plates at a density of 1 × 10^4^/well, were exposed to conditioned or non-conditioned medium (NCM) for 24 h at 37°C, followed by infection with TcCOL-NLuc trypomastigotes (5 parasites/cell). Sixteen hours post-infection (*T* = 0), cells were washed three times with PBS to remove non-internalized parasites and incubated in either CM or NCM. The medium (conditioned or non-conditioned) was replenished at 48 h post-infection. Parasite growth was determined at 0 (*T* = 0), 1 (*T* = 1), and 3 (*T* = 3) days post-infection by measuring NLuc activity (RLU) normalized within each well to live-cell numbers (RFU) as described above.

### Statistical Analysis

All bar graphs were displayed as means ± SD. In box and whisker plots, boxes represent first and third quartiles with a line at the median and whiskers indicate the maximum and minimum data values. Statistical analysis of differences between mean values of groups was determined by the unpaired two-tailed Student’s *t*-test, using GraphPad Prism software version 8.3.1. A *p*-value < 0.01 was considered significant.

## Results

### 3D Cultures of JEG-3 Cells Display Differentiation Characteristics of Human Placenta Trophoblasts

We established a 3D culture of trophoblast-derived JEG-3 cells associated with human brain microvascular endothelial cells [HBMECs; (Coyne et al., 2007)] in a rotating wall vessel (RWV) bioreactor (Unsworth and Lelkes, 1998; Barrila et al., 2010; McConkey et al., 2016). To initiate the culture, HBMECs were incubated with collagen-coated microcarrier beads and inoculated into a slow-turning lateral vessel attached to the RWV platform (Supplementary Figure 1). After 3 days of incubation under slow rotation, we added JEG-3 cells and continued the culture for 17 more days. Cell-coated beads (spheroids) could be observed by fluorescence microscopy after Texas Red^®^ DHPE and DAPI staining (Figure 1A). Approximately 85% of the beads were covered with cells under these conditions.

To determine whether 3D JEG-3 cells display characteristics of native placental trophoblasts, such as cell fusion and multinucleated morphology, 3D grown spheroids (harvested at day 20 of culture) were fixed, permeabilized and stained with Alexa Fluor^®^ 680 phalloidin to visualize actin filaments (Figure 1B). We found that cells grown in the RWV bioreactor fused and formed large syncytia that could be observed either after being detached from the beads and transferred to slides (Figure 1B, middle panel) or directly at the spheroid’s surface (Figure 1B, right panel), as described in previous studies (McConkey et al., 2016; Corry et al., 2017). In contrast, no multinucleated cells were observed in 2D cultured JEG-3 cells (Figure 1B, left panel).

To further characterize JEG-3 cells grown in 3D, we confirmed their expression of cytokeratin-19 (CYT-19), a cytoskeleton protein that is a specific marker of epithelial cells differentiation (Figure 2A; Aplin et al., 2009). An immunofluorescence assay showed positive CYT-19 staining of 2D cultured JEG-3 cells (top panels) and 3D-grown JEG-3 cells, either when covering spheroids (lower panels) or after being detached from the cell-coated beads (3rd panels from top). No CYT-19 staining was detected in HBMECs (2nd panels from top).

Similar results were obtained when 3D-grown cultures were evaluated by flow cytometry (Figure 2B). Two distinct populations were observed in cells harvested from 3D JEG-3 spheroids (top panels). Based on CYT-19 staining of 2D JEG-3 and HBMEC cells (middle and lower panels, respectively), we identified one population positive for this marker, comprising about 65% of the live cells, which represents 3D-JEG-3 cells (top right panel). The second population tested negative for CYT-19 and in agreement with the results shown in Figure 2A, corresponds to HBMECs. Consistent with previous findings (McConkey et al., 2016; Corry et al., 2017), our results suggest that most of the cells coating the beads (after 20 days in our 3D culture system) are JEG-3 cells.

### 3D-Cultured JEG-3 Cells Express Markers Associated With Syncytiotrophoblasts’ Differentiation

Syncytiotrophoblasts play crucial roles during pregnancy, including synthesis of specific hormones that regulate embryo implantation, fetal development and maternal metabolism (Costa, 2016).

**FIGURE 3:**
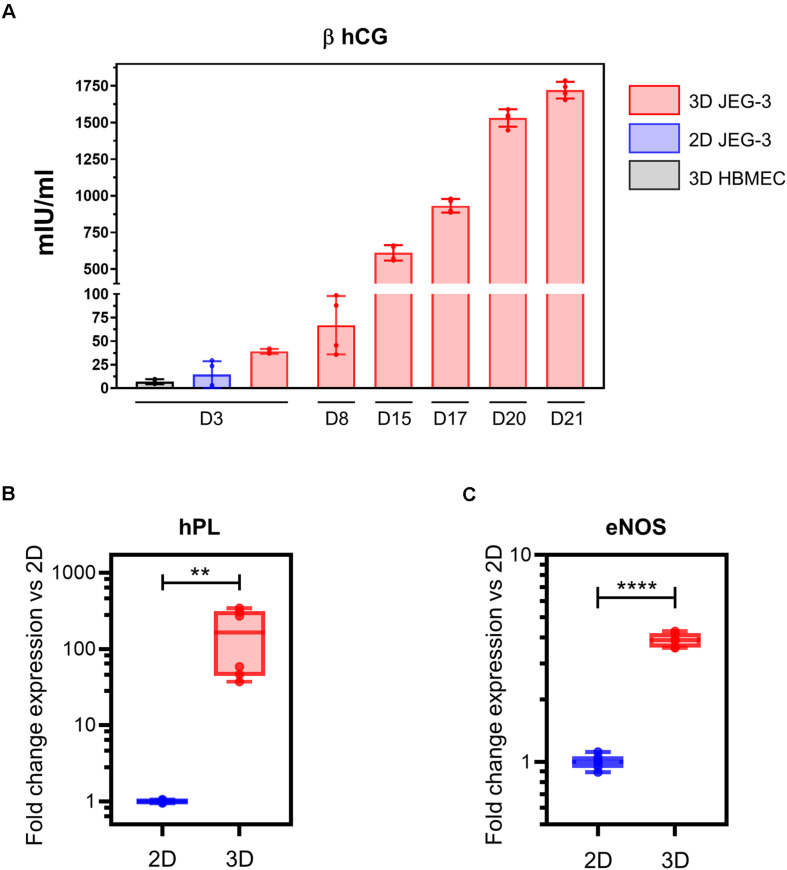
3D-cultured JEG-3 cells express markers associated with syncytiotrophoblasts’ differentiation. **(A)** ELISA assay for β human chorionic gonadotrophin (β hCG) from culture supernatants of 2D JEG-3 cells, 3D HBMECs and 3D-grown spheroid cultures, as indicated. Data represent the mean ± SD of two independent vessels or 2D cultures with samples tested in duplicates. **(B)** qRT-PCR analysis of human placental lactogen (hPL) mRNA from two independent vessels of spheroid cultures (3D) or conventional flasks of JEG-3 cells (2D). Fold change in gene expression was determined by the 2 ^–ΔΔ*Ct*^ method with samples normalized to β actin. Boxes represent first and third quartiles with a line at the median. Whiskers indicate maximum and minimum data values. **(C)** qRT-PCR analysis of endothelial nitric oxide synthase (eNOS) mRNA from two independent vessels of spheroid cultures (3D) or conventional flasks of JEG-3 cells (2D). Fold change in gene expression calculated and plotted as in **(B)**. In all panels, asterisks indicate statistically significant differences calculated by the unpaired *t*-test. **P ≤ 0.01; ****P ≤ 0.0001.

To evaluate whether our 3D JEG-3 cultures released beta human chorionic gonadotropin (β hCG), we tested culture supernatants collected at different time points throughout the 21 day culture period by ELISA (Figure 3A). Beta hCG was detected in culture supernatants of 3D JEG3 cells at all-time points tested starting at day 3. Levels increased over time up to 1,750 mIU/ml measured at day 21. No significant levels of hormone were detected at day 3 in either culture supernatants of HBMECs grown on beads under 3D conditions (3D HBMEC) and used as control, or in culture supernatants of JEG-3 cells grown in conventional cultures (2D JEG-3). Beta hCG was not evaluated at later time points for these two cell lines because (a) 3D HBMECs serve as scaffolds and therefore do not grow under optimum conditions in 3D JEG-3 cultures, and (b) 2D JEG-3 cells are not used in any experiment after 3 days of culture. These results show that 3D JEG-3 cells release β hCG when grown in this culture system similarly to primary trophoblast cells isolated from term placentas as reported previously (Li and Schust, 2015).

**FIGURE 4:**
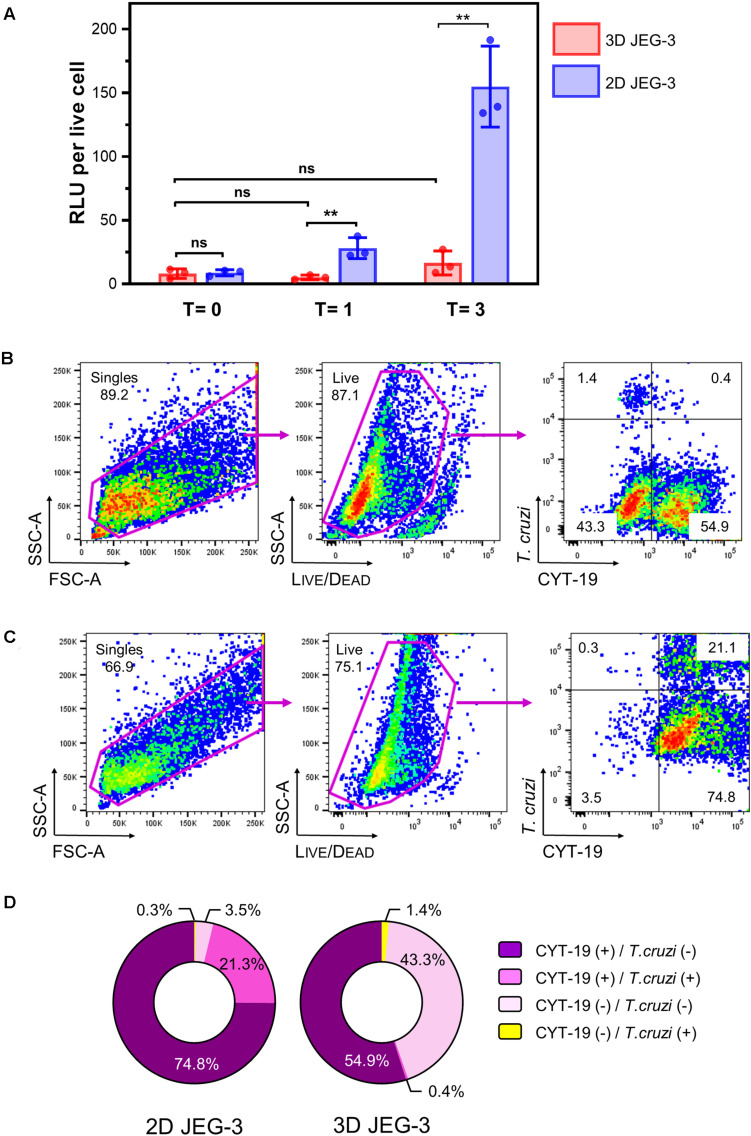
3D-grown JEG-3 cells resist *T. cruzi* infection. **(A)** Monolayers of JEG-3 cells (2D JEG-3) grown in 96-well plates and spheroids (3D JEG-3) transferred from the RWV bioreactor to 96-well plates, were infected with *T. cruzi-*luminescent parasites at a ratio of 5 parasites per live cell. The infection was conducted under static conditions and monitored over time by measuring intracellular nanoluciferase activity at 0 (*T* = 0), 1 (*T* = 1), and 3 (*T* = 3) dpi. Luminescence was quantitated in relative light units (RLU) and normalized to the number of live cells. Data represent the mean ± SD of three technical replicates and is representative of 3 independent experiments. Asterisks indicate statistically significant differences calculated by an unpaired *t*-test. **P ≤ 0.01; ns: non-significant. **(B)** 3D JEG-3 cells were infected with CellTrace^TM^ Far Red- labeled trypomastigotes at a ratio of 5 parasites per live cell under slow rotation in the RWV bioreactor. Sixteen hours post-infection, cells were detached from the beads, stained for CYT-19 and analyzed by flow cytometry. The gating strategy used to identify singles and exclude dead cells (based on LIVE/DEAD^TM^ violet dye staining) is shown. Data are representative of two independent experiments. **(C)** Same as in **(B)** but infection was conducted in 2D cultured JEG-3 cells. **(D)** Pie charts illustrating the distribution of cell populations from 2D and 3D JEG-3 cells according to infectivity and CYT-19 status.

Next, we assessed the expression of hPL mRNA in 2D and 3D JEG-3 cells by quantitative RT-PCR using β actin as housekeeping gene. This analysis revealed that hPL mRNA was up-regulated approximately 300-fold in 3D JEG-3 cells compared to 2D JEG-3 cells (Figure 3B). Our findings are consistent with previous studies showing overexpression of hPL mRNA in JEG-3 cells cultured in the RWV bioreactor, and mimic the secretion profile of primary trophoblast cells (McConkey et al., 2016). No hPL mRNA was detected in total RNA extracted from HBMECs (Supplementary Figure 2A).

To further study the expression profile of placental differentiation markers in 3D cultured spheroids, we measured the levels of endothelial nitric oxide synthase (eNOS), an enzyme that participates in nitric oxide synthesis and thus, is associated with oxidative stress. eNOS mRNA was expressed at approximately fourfold higher levels in 3D-cultured JEG-3 cells compared to 2D (Figure 3C), in agreement with former reports which showed its overexpression in differentiated STs (Lyall et al., 1998). No eNOS mRNA was detected in total RNA extracted from HBMECs (Supplementary Figure 2B).

Together, our data (Figures 1–3) indicate that 3D-cultured JEG-3 cells exhibit differentiation characteristics comparable to *in vivo* human syncytiotrophoblasts such as formation of syncytia, production of β-human chorionic gonadotrophin and up-regulation of hPL and eNOS mRNA.

### 3D JEG-3 Cells Are Refractory to *Trypanosoma cruzi* Infection

To investigate whether *T. cruzi* trypomastigotes were able to infect and replicate in 3D-grown JEG-3 cells, we used a stable transgenic cell line of parasites expressing nanoluciferase [TcCOL-NLuc, (Silberstein et al., 2018)] in order to follow the infection over time. Preliminary experiments showed that nanoluciferase activity normalized to the number of viable cultured cells could be used as a measure of parasite growth. As shown in Supplementary Figure 3, infection of LLC-MK2 or HBMEC cells with TcCOL-NLuc trypomastigotes resulted in an increase (45.2-fold for LLC-MK2 and 99.8-fold for HBMEC) in luminescence per live-cell over a 3 day period.

We used this methodology to compare the susceptibility of 2D and 3D-grown JEG-3 cells to TcCOL-NLuc infection under static conditions. JEG-3 cells grown either in 2D conditions in 96-well plates or in 3D conditions in a RWV bioreactor vessel (in which case, day 20 spheroids were removed from the RWV bioreactor and transferred to 96-well plates), were infected at a ratio of 5 parasites per live cell as described in the methods section. Sixteen hours post-infection (16 hpi), cultures were washed to remove non-internalized parasites, and the infection proceeded for 3 additional days. Nanoluciferase activity was measured at 16 hpi (T = 0), and subsequently at 1 (T = 1) and 3 (T = 3) days post-infection (dpi). We found that TcCOL-NLuc parasites infected and multiplied in JEG-3 cells cultured in conventional 2D conditions, as indicated by the 18.56 -fold increase in the nanoluciferase activity (RLU/live cell) in 3 days (Figure 4A, blue bars). In contrast, TcCOL-NLuc infection of 3D JEG-3 cells was notably impaired since the nanoluciferase activity did not significantly change overtime (Figure 4A, red bars).

Although we showed that the spheroids’ surface was mainly covered by layers of large multinucleated JEG-3 cells (Figure 1B), suggesting they are fully accessible to *T. cruzi* invasion, it was important to determine whether parasites could also infect HBMECs within the spheroids that were used as scaffolds to initiate the 3D cultures. To that end, spheroids were infected in the bioreactor’s vessel under slow rotation with CellTrace^TM^ Far Red-labeled trypomastigotes, to facilitate parasite tracking and detection by flow cytometry. Cells were harvested 16 hpi and stained for CYT-19 to identify JEG-3 cells (Figure 2). Under this infection environment, we observed that the majority (55.3%) of the live cells were positive for CYT-19 (Figure 4B, right panel, upper, and lower right quadrants) and of those, only 0.4% were infected with *T. cruzi* (Figure 4B, right panel, upper right quadrant). For comparison, under conventional 2D culture and static infection conditions, we observed that 21.1% of live CYT-19 positive 2D JEG-3 cells were infected with *T. cruzi* (Figure 4C, right panel, upper right quadrant).

It is important to note that fluid shear stress, resulting from the slow rotation of the RWV (Figure 4B), can affect parasite infection as illustrated by the reduced susceptibility (approximately 6.5-fold) to *T. cruzi* infection exhibited by HBMECs grown in 3D compared to HBMECs grown in conventional 2D cultures (Supplementary Figure 5). Taking into consideration this ∼6.5-fold reduction in parasite infection when done under rotation, the percent of infected 3D JEG-3 cells remains significantly lower (eightfold lower) than the percent of infected 2D JEG-3 cells (Figure 4, panels B,C). This flow cytometry experiment also revealed that 1.4% of the live CYT-19 negative cells, i.e., HBMECs, were infected at 16 hpi (Figure 4B, right panel, upper left quadrant), suggesting that *T. cruzi* can infect HBMECs in 3D JEG-3 spheroids and contribute to the nanoluciferase activity measured after 3 days of infection, as detected in Figure 4A.

Taken together, our results show that *T. cruzi* parasite infection is severely restricted in JEG-3 cells grown as spheroids in 3D conditions.

### 3D Cultures of JEG-3 Cells Release Paracrine Factors That Prevent *Trypanosoma cruzi* Infection

**FIGURE 5:**
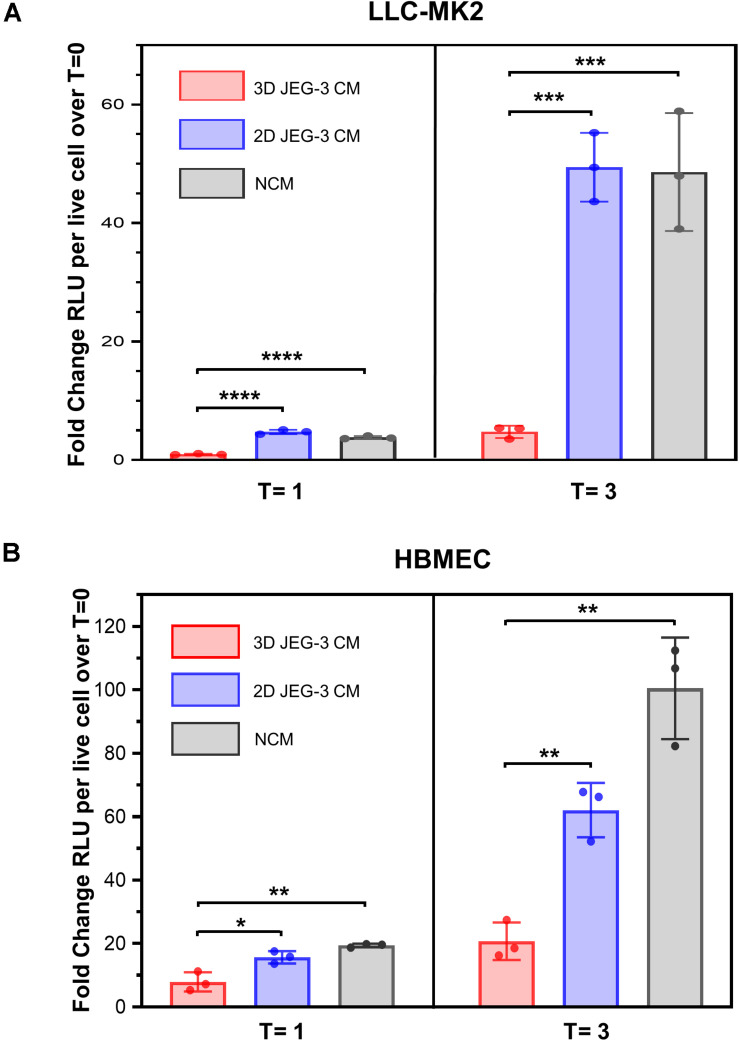
3D cultures of JEG-3 cells release paracrine factors that prevent *T. cruzi* infection of non-trophoblastic cells. **(A)** LLC-MK2 or **(B)** HBMEC cells were preincubated with supernatants from 2D JEG-3 (2D CM), 3D JEG-3 (3D CM) or non-conditioned medium (NCM) (LLC-MK2 or HBMECs culture medium) for 24 h followed by infection with *T. cruzi-*luminescent parasites (5 parasites per live cell). Nanoluciferase activity was measured as relative luminescence units (RLU) at 0 (*T* = 0), 1 (*T* = 1), and 3 (*T* = 3) dpi. Data were normalized to the number of live cells and represent the mean ± SD of three technical replicates. The fold change RLU values per live cell over T = 0 were plotted. The experiment was repeated two times with similar results. In all panels, asterisks indicate statistically significant differences calculated using an unpaired *t*-test. *P ≤ 0.05; **P ≤ 0.01; ***P ≤ 0.001; ****P ≤ 0.0001.

It was reported that primary human trophoblasts pass on viral resistance to non-trophoblastic cells by secretion of antiviral factors (soluble or contained in exosomes) to the culture media (Bayer et al., 2018). Hence, we next investigated whether 3D-cultured JEG-3 cells released factors that would impact *T. cruzi*’s ability to invade and grow in other mammalian cells besides trophoblasts. LLC-MK2 and HBMEC cells, both susceptible to *T. cruzi* infection (Supplementary Figure 3), were pre-exposed to culture supernatants (conditioned media, CM) of 3D or 2D-cultured JEG-3 cells for 24 h and subsequently infected with TcCOL-NLuc trypomastigotes and maintained in CM. Parasite infection was quantified at 16 hpi (T = 0), 1 dpi (T = 1), and 3 dpi (T = 3) by measuring nanoluciferase activity (RLU) per live cell. The fold change in RLU at T = 1 and T = 3 compared to T = 0, representing parasite growth in these cultures, is shown in Figure 5. At T = 3, we observed a 10-fold reduction in parasite growth in LLC-MK2 cells that were exposed to 3D JEG-3 cells conditioned media (3D CM) compared to cells exposed to non-conditioned media (NCM), used as control, or exposed to 2D JEG-3 cells conditioned media (2D CM) (Figure 5A). This inhibitory effect could be observed at 1 and 3 dpi, although the reduction in parasite growth was more evident at 3 dpi (Figure 5A). Likewise, parasite growth was reduced by fivefold in HBMECs exposed to 3D CM (Figure 5B). Notably, a reduction in cell viability was observed when LLC-MK2 and HBMEC cells were expose to conditioned media (Supplementary Figure 4). The effect was more obvious in LLC-MK2 cells (Supplementary Figure 4A) in which the percent of viable cells diminished to 50% when cells were incubated in 3D CM compared to NCM control. Since *T. cruzi* infectivity was measured per intact viable cell (Niles et al., 2007), this decrease in cell viability does not influence our observation that preincubation with 3D CM significantly reduces the growth of *T. cruzi* in both cell types.

Consistent with the known intrinsic resistance of the human placenta to pathogen infections (Corry et al., 2017; Bayer et al., 2018), our results suggest that JEG-3 cells cultured in 3D release paracrine factors that protect them and other non-trophoblastic cells from *T. cruzi* infection, highlighting the value of this model to study host-parasite interactions.

## Discussion

In recent years, *Trypanosoma cruzi* vertical transmission has become a globalized public health challenge that is contributing to perpetuate CD in endemic and non-endemic countries. With the implementation of successful blood screening strategies which significantly reduced the risk of transfusion transmitted infections, congenital transmission is now considered an important mode of CD spread in non-endemic countries where no routine testing of pregnant women for the disease is implemented (Antinori et al., 2017).

The main cellular mechanisms that lead to fetal infection by *T. cruzi*, despite the presence of a placental barrier, are still under investigation. Finding specific molecules and pathways involved in parasite invasion and replication in placental cells is essential to better understand the complexity of congenital infections. Mother-to-child transmission of CD most likely occurs when bloodstream trypomastigotes reach the placental intervillous space and interact with the large cellular surface provided by the syncytioptrophoblasts (Blaszkowska and Goralska, 2014). These highly specialized cells not only function as a physical barrier between mother and fetus, but also modulate immune responses against pathogen infections (Arora et al., 2017; Ander et al., 2019) and thus, constitute an attractive setting for studying *T. cruzi*-placenta interactions.

In this study, as a first step toward better understanding the cellular mechanisms involved in parasite vertical transmission, we report that *T. cruzi* infection is severely restricted in STs by using a 3D culture model system of human JEG-3 trophoblasts. The organotypic model we used is based in the co-culture of JEG-3 cells with HBMECs which are initially employed to cover Cytodex-beads and facilitate trophoblasts’ attachment (McConkey et al., 2016; Corry et al., 2017).

Three-dimensional culture of JEG-3/HBMEC spheroids promoted JEG-3 cells differentiation revealed by the formation of syncytia (Figure 1B) and production of β human chorionic gonadotropin and hPL (Figures 3A,B), two key features of human syncytiotrophoblasts mimicking the secretory profiles observed in trophoblast cells isolated from term placentas (Li and Schust, 2015). In contrast, JEG-3 cells cultured in conventional tissue culture flasks did not form syncytia and produced small amounts of placenta-specific hormones, which highlights the value of this 3D model to represent the local microenvironment where cell architecture and functions are being recreated.

Further analysis of the 3D-grown spheroids showed that the beads were mostly covered by JEG-3 cells as indicated by their positive staining for cytokeratin-19, a specific marker of epithelial cells as illustrated in Figures 2A,B. We routinely observed that the spheroids contained approximately 65% of CYT-19 positive cells, which is slightly lower than the 75% estimate reported by others (McConkey et al., 2016). The reason for this difference could be attributed to variations in the overall culture conditions across laboratories, such as slight fluctuations in nutrients’ and growth factors’ availability.

Using *T. cruzi* trypomastigotes of the Colombiana strain expressing nanoluciferase (Silberstein et al., 2018), and through a combination of cell-based activity assays and flow cytometry analysis, we evaluated parasite infection overtime in 3D-grown spheroids. *T. cruzi* infection was monitored by assessing nanoluciferase activity in infected cells. Although indirect, luminescence produced by transgenic parasites expressing a luciferase reporter has been shown to be a reliable measure of parasite growth *in vitro* (Lang et al., 2005; Lewis et al., 2014). *T. cruzi* grew efficiently in 2D JEG-3 cells, as represented by an 18-fold increase in the luminescence signal per viable cell over 3 days following infection with TcCOL-NLuc parasites (Figure 4A). In contrast, the luminescence signal did not significantly increase in 3D-grown cells (spheroids transferred to plates and infected under the same static conditions), suggesting the resistance of 3D JEG3 cells to *T. cruzi* infection (Figure 4A).

Because we observed that the spheroids contained two distinctive cell populations based on CYT-19 staining (Figure 2B, top panels), it was important to identify which cells were infected and responsible for the nanoluciferase activity detected in infected spheroids. Flow cytometry analysis of 3D-cultured spheroids infected under 3D culture conditions with labeled parasites, revealed that 0.4% of the 3D JEG-3 cells, and 1.4% of the HBMECs were infected (Figure 4B). These results showed that infected HBMECs and not JEG-3 cells were likely responsible for the nanoluciferase activity detected in the spheroids (Figure 4A). Together, the small level of infectivity detected by luminescence and the scarce percentage of parasitized cells, indicate that 3D-cultured JEG-3 cells most probably activate intrinsic defense mechanisms to prevent *T. cruzi* infection. Our results are consistent with previous reports which have shown that 3D JEG-3 cells cultured in a similar system, resist infection by vesicular stomatitis virus, Zika virus and *Toxoplasma gondii* (McConkey et al., 2016; Corry et al., 2017). However, the molecular processes involved in 3D-JEG3 cells’ resistance to *T*. *cruzi* infection, which may involve parasite invasion, differentiation and/or intracellular amastigotes multiplication, remain to be elucidated.

We found that rotation affects *T. cruzi* infection of 3D cultured cells (Supplementary Figure 5). It has been shown that the RWV bioreactor system induces physiological levels of fluid shear stress that mimic the environment encountered by pathogens in the infected host (Nauman et al., 2007). We speculate that the 3D JEG-3 cells culture system may reproduce the fluid movement around the floating placenta microvilli impacting the ability of *T. cruzi* to infect the spheroids in a 3D culture environment. However, fluid sheer stress alone would not account for the 89% reduction in infection of 3D vs. 2D grown cells observed in our studies (Figure 4A), which supports activation of intrinsic mechanisms of resistance in 3D-grown JEG-3 cells. Moreover, our model might closer represent the host natural setting, where the parasite is vertically transmitted only to around 5% of the children born from infected mothers (Carlier et al., 2019).

The human placenta functions is an immuno-modulatory organ, and as such, expresses cytokines, chemokines and high levels of eNOS, that generates nitric oxide (NO), and a state of oxidative stress (Myatt and Cui, 2004). Production of eNOS plays an important role in trophoblasts’ function and differentiation (Lyall et al., 1998). Similarly, we found that eNOS mRNA expression was up-regulated in 3D-grown JEG-3 cells (Figure 3C) which also correlates with recent work conducted in chorionic villi explants (Triquell et al., 2018). Whether NO is released upon infection, or its role in restricting the growth of *T. cruzi* in these cultures, remains to be determined.

The potential placental defense mechanisms against blood trypomastigotes have been previously investigated. For instance, it has been shown that *T. cruzi* induces tissue disorganization and destruction of chorionic villi *ex vivo* (Duaso et al., 2010) and in placentas from women with CD (Duaso et al., 2012). Others have found that the continuous renewal of the trophoblast epithelia leads to parasite clearance (Liempi et al., 2014, 2016; Droguett et al., 2017). In addition, Toll-like receptors (TLRs) expressed at the cell surface of human trophoblasts or in endosomes (Koga et al., 2014), recognize *T. cruzi*-associated molecular patterns (Tarleton, 2007; Pellegrini et al., 2011; Castillo et al., 2017a,b) which in turn trigger production of inflammatory cytokines and type I interferons (Kawai and Akira, 2010). Transcriptome profiling studies comparing 2D and 3D JEG-3 cells exposed to *T. cruzi* parasites will be helpful to identify potential specific molecular mechanisms that render 3D-grown trophoblasts resistant to infection. Such studies may reveal protein networks associated with either parasite invasion, differentiation and/or intracellular multiplication and help better understand how parasites may circumvent these mechanisms resulting in vertical transmission events.

Lastly, and consistent with former reports (Corry et al., 2017; Bayer et al., 2018; Levine et al., 2020), we observed that 3D-cultured JEG-3 cells released factors that prevented *T. cruzi*’s growth in non-trophoblast mammalian cells (Figure 5). It is well documented that secreted type III interferons mediate trophoblast’s protection from Zika virus infection (Chen et al., 2017; Corry et al., 2017) and that STs respond to *Toxoplasma gondii* infection by producing immunomodulatory chemokines that limit parasite growth at the level of attachment and replication (Ander et al., 2018). Likewise, it was reported that chromosome 19 microRNAs contained in exosomes released by trophoblasts, are involved in antiviral activity (Bayer et al., 2018). Based on those findings, we speculate that the molecules present in culture supernatants of 3D-grown JEG-3 cells, could potentially be immuno-modulatory proteins or miRNA-containing extracellular vesicles released by JEG-3 cells upon parasite stimulation. Future proteomic analysis and miRNA profiling studies will facilitate the discovery of factors secreted by trophoblasts that block *T. cruzi* infection of STs and other cells.

Research on *T. cruzi* vertical transmission is currently limited because of ethical reasons. Our work validates the use of 3D-grown JEG-3 spheroids to study the processes by which trypomastigotes bypass the human placental barrier. A better understanding of the mechanisms involved in *T. cruzi* congenital infection may open new opportunities for the identification and evaluation of therapeutics to reduce congenital CD. Similarly, this 3D culture system could be exploited to better understand the role played by JEG-3 cells in controlling other vertically transmitted infections.

## Data Availability Statement

The original contributions presented in the study are included in the article/Supplementary Material, further inquiries can be directed to the corresponding author/s.

## Ethics Statement

The experiments using the recombinant strain of *T. cruzi* Colombiana expressing nanoluciferase (TcCOL-NLuc) were approved by the Food and Drug Administration White Oak Institutional Biosafety Committee under protocol number IBC#10192.

## Author Contributions

ES designed the research, conducted the experiments, performed data analysis, wrote, reviewed, and edited the manuscript. KK provided the HBMEC cells and edited the manuscript. DA provided technical support. AD designed and supervised the research, performed data analysis, wrote, reviewed, and edited the manuscript. All authors contributed to the article and approved the submitted version.

## Conflict of Interest

The authors declare that the research was conducted in the absence of any commercial or financial relationships that could be construed as a potential conflict of interest.
