# Supplementary material for: Human Placental Trophoblasts Are Resistant to Trypanosoma cruzi Infection in a 3D-Culture Model of the Maternal-Fetal Interface
Source: Front Microbiol. 2021 Mar 4;12:626370. doi: 10.3389/fmicb.2021.626370 (PMC7969514; doi:10.3389/fmicb.2021.626370)
Supplement: Supplementary file 1 [file Data_Sheet_1.docx]

Supplementary Material

## Supplementary Figures

**
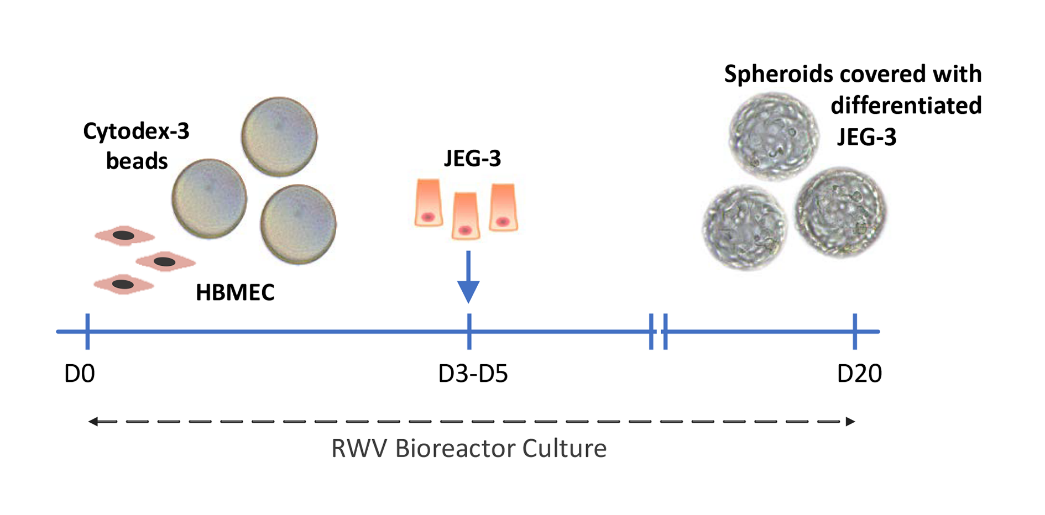
**

**Supplementary Figure 1.** Schematic representation of the generation of JEG-3 cell-covered spheroids. Human brain microvascular endothelial cells (HBMECs) resuspended in GTSF-2 media were mixed with 50 mg collagen-coated beads (Cytodex-3 beads). After a 30 minutes incubation at 37°C, the cell/bead slurry was transferred to a disposable slow-turning lateral 10 ml culture vessel and attached to the rotary wall vessel (RWV) bioreactor. After 3 days, JEG-3 cells were added, and the 3D culture continued for 17 additional days.


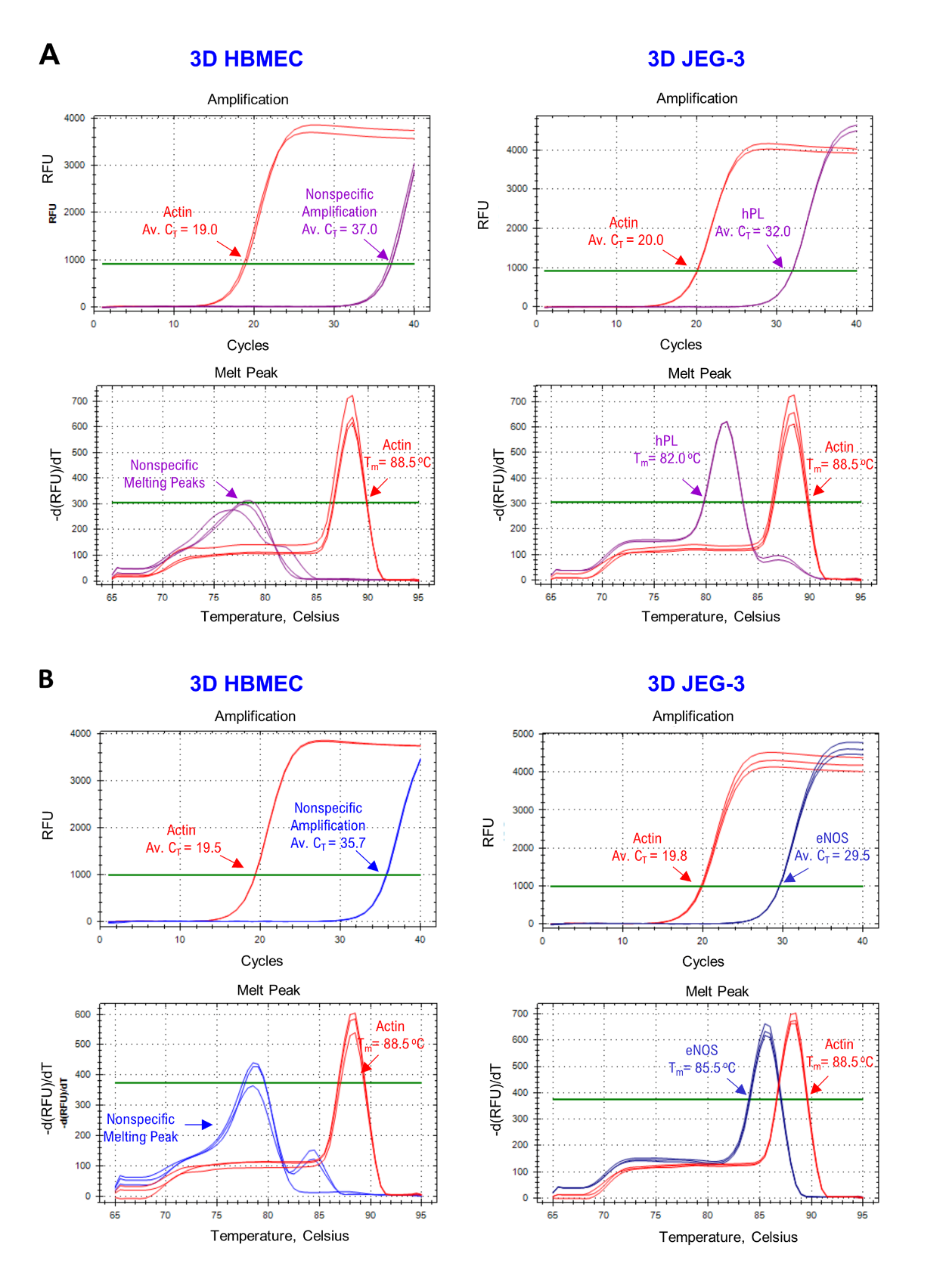


**Supplementary Figure 2.** qRT-PCR evaluation of total RNA extracted from 3D HBMEC or 3D JEG-3 cells. **(A)** Human placental lactogen and β actin. **(B)** eNOS and β actin**.** Figures show representative amplification plots and melt curve analysis of reaction products. Cycle thresholds (C_T_) value for beta actin, hPL, and eNOS are indicated. hPL and eNOS mRNAs are not detected in 3D HBMECs.

**
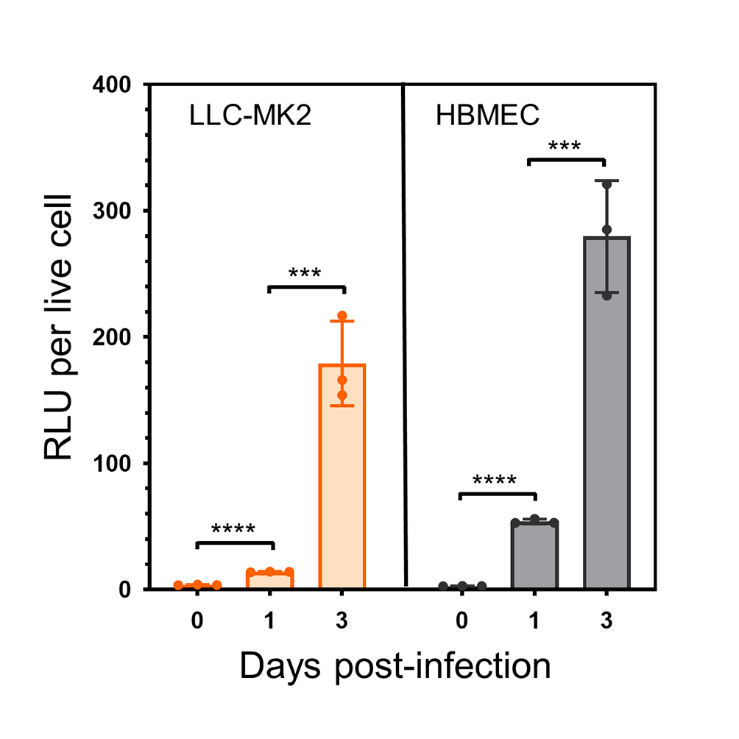
**

**Supplementary Figure 3.** Quantification of *T. cruzi* parasites in LLC-MK2 and HBMEC cells by luminescence. Monolayers of LLC-MK2 and HBMEC cells were infected with *T. cruzi-*luminescent trypomastigotes (TcCOL-NLuc) at a ratio of 5 parasites per live cell. The infection was conducted under static conditions and monitored over time by measuring intracellular nanoluciferase activity at 0 (T=0), 1 (T=1) and 3 (T=3) days post infection. Luminescence was quantitated in relative light units (RLU) and normalized to the number of live cells. Data represent the mean ± SD of three technical replicates. The experiment was repeated three times with similar results. Asterisks indicate statistically significant differences calculated by an unpaired t-test**.** ***: P ≤ 0.001; ****: P ≤ 0.0001.


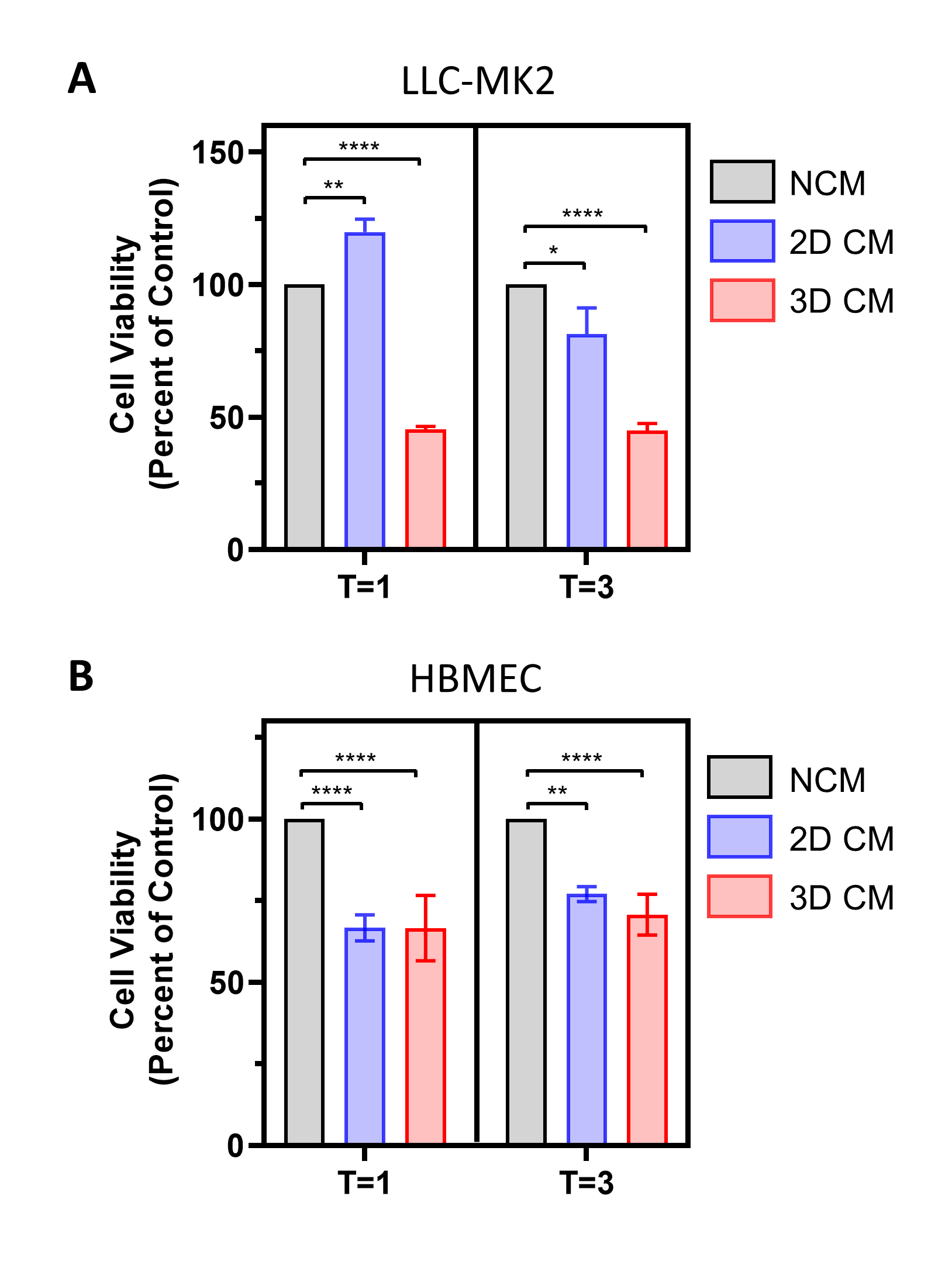


# Supplementary Figure 4. Effect of conditioned medium on the viability of LLC-MK2 and HBMEC cells. Cell viability was measured using the CellTiter-Fluor™ Cell Viability Assay. (A) LLC-MK2 or (B) HBMEC cells were incubated with culture supernatants, i.e conditioned medium (CM) from 2D JEG-3 (2D CM), 3D JEG-3 (3D CM) or non-conditioned medium (NCM; LLC-MK2 or HBMECs growth medium, as appropriate). Relative fluorescence units were determined at 0 (T=0), 1 (T=1) and 3 (T=3) days in culture. Cell viability is plotted as percent of control (NCM).


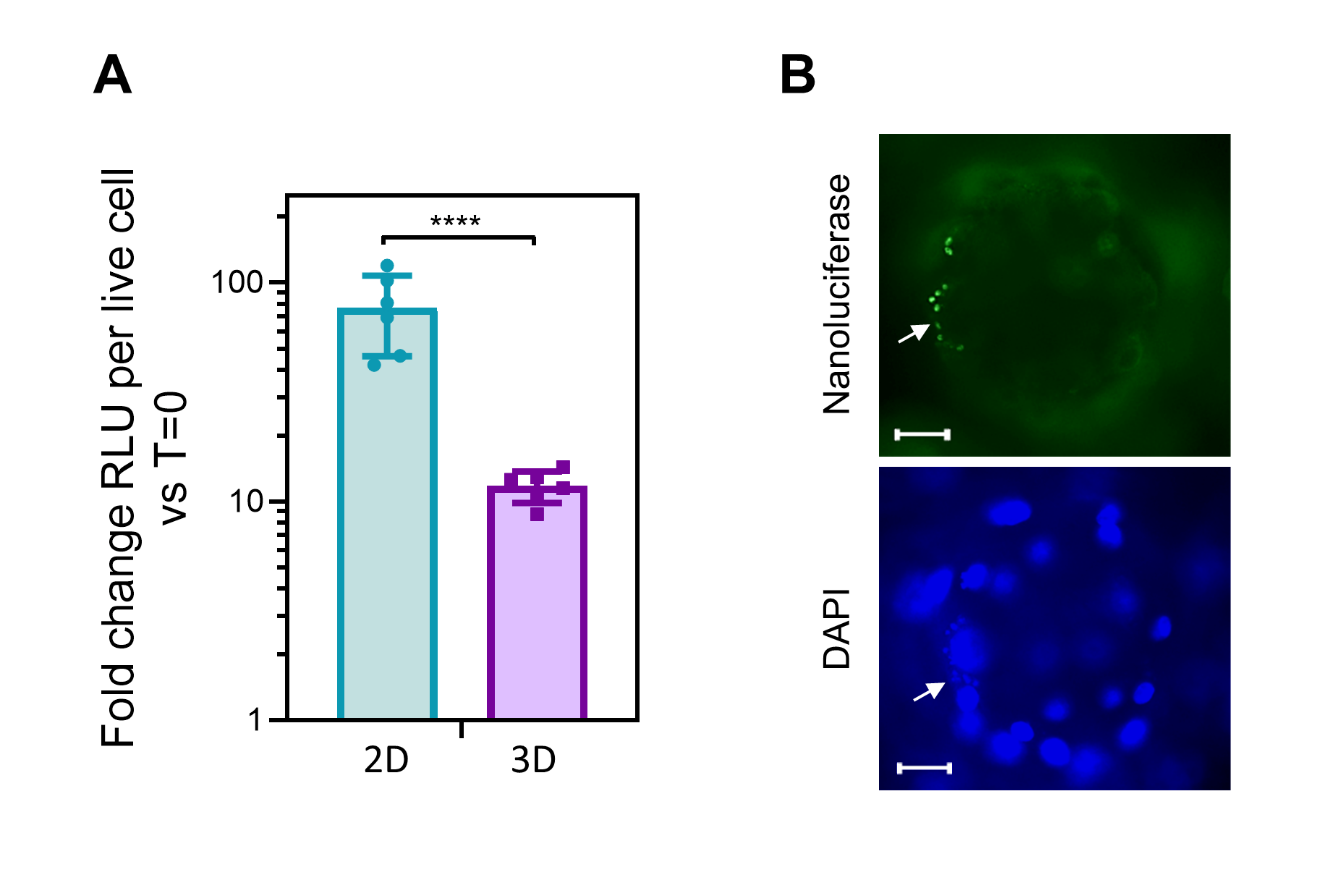


# Supplementary Figure 5. *T. cruzi* infection under static (2D) or rotating (3D) conditions of HBMECs. (A) Monolayers of HBMEC cells (2D) grown in 96-well plates and 3D HBMEC grown on spheroids (3D) were infected with *T. cruzi-*luminescent parasites (TcCOL-NLuc) at a ratio of 5 parasites per live cell. The infection was conducted under static conditions for 2D HBMECs and under slow rotation for 3D HBMECs. Sixteen hours post-infection, cells were washed to remove non-internalized parasites. Three days post-infection, 3D HBMEC spheroids were removed from the RWV bioreactor’s vessel and transferred to 96-well plates (100 μl/well). Parasite growth was determined in 2D and 3D infected HBMECs by measuring intracellular nanoluciferase activity at 0 (T=0) and 3 (T=3) dpi. Luminescence was quantitated in relative light units (RLU) and normalized to the number of live cells. Data represent the mean ± SD of two independent experiments, with three technical replicates each. The fold change RLU values per live cell over T=0 were plotted. Asterisks indicate statistically significant differences calculated by an unpaired t-test. ****: P ≤ 0.0001. (B) 3D infected HBMEC spheroids were fixed/permeabilized on day 3 post-infection and stained with rabbit anti-nanoluciferase (1/200, Promega, WI) followed by Alexa Fluor® 488 anti-rabbit IgG (H+L) antibodies (green). Slides were mounted with Vectashield mounting medium containing DAPI to visualize nuclei (blue). Fluorescent micrographs were acquired on a Keyene BZ-9000 fluorescence microscope. Arrows point to *T. cruzi* amastigotes. Scale bar: 30 μm.
